# Ongoing complete response after treatment cessation with dabrafenib, trametinib, and cetuximab as third-line treatment in a patient with advanced BRAF^V600E^ mutated, microsatellite-stable colon cancer: A case report and literature review

**DOI:** 10.3389/fonc.2023.1166545

**Published:** 2023-05-05

**Authors:** Gudrun Piringer, Jörn Decker, Vera Trommet, Thomas Kühr, Sonja Heibl, Konrad Dörfler, Josef Thaler

**Affiliations:** ^1^ Department of Internal Medicine IV, Wels-Grieskirchen Medical Hospital, Wels, Austria; ^2^ Department of Hematology and Oncology, Kepler University Hospital, Linz, Austria; ^3^ Medical Faculty, Johannes Kepler University Linz, Linz, Austria; ^4^ Department of Internal Medicine, Klinikum Rohrbach, Rohrbach, Austria

**Keywords:** combination targeted therapy, dabrafenib, trametinib, MSS, BRAF-V600 mutation

## Abstract

Metastatic BRAF^V600E^ mutated colorectal cancer is associated with poor overall survival and modest effectiveness to standard therapies. Furthermore, survival is influenced by the microsatellite status. Patients with microsatellite-stable and BRAF^V600E^ mutated colorectal cancer have the worst prognosis under the wide range of genetic subgroups in colorectal cancer. Herein, we present a patient case of an impressive therapeutic efficacy of dabrafenib, trametinib, and cetuximab as later-line therapy in a 52-year-old woman with advanced BRAF^V600E^ mutated, microsatellite-stable colon cancer. This patient achieved a complete response after 1 year of triple therapy. Due to skin toxicity grade 3 and recurrent urinary tract infections due to mucosal toxicity, a therapy de-escalation to dabrafenib and trametinib was performed, and the double therapy was administered for further 41 months with ongoing complete response. For 1 year, the patient was off therapy and is still in complete remission.

## Introduction

BRAF is a component of the RAS-RAF-MAPK signaling pathway (1). Eight to 12% of metastatic colorectal cancer (CRC) and approximately half of the patients with melanoma have a BRAF mutation (2). BRAF*^V600E^* mutation is the most frequent BRAF mutation (90%) and leads to constitutive, RAS-independent activation of BRAF kinase activity and MAPK pathway signaling through downstream activation of MEK (MEK 1 and MEK 2) and ERK (ERK1 and ERK2) kinases and promotes tumor cell migration, proliferation, and survival (2, 3). In metastatic CRC, BRAF*^V600E^* mutation is associated with right-side, poorly differentiated, and mucinous-type tumors and is a negative prognostic factor (4). Its mortality is a nearly twofold increase compared to that of BRAF wild-type tumors (5) due to poor response to standard therapies (5–7).

Several studies investigated the effect of targeted therapies in BRAF*^V600E^* mutated tumors to improve the outcome. Encorafenib, dabrafenib, and vemurafenib are potent tyrosine kinase inhibitors of the BRAF*^V600E^* kinase, and trametinib and binimetinib potently inhibit the MEK kinase, although BRAF or MEK inhibitor monotherapy showed dramatic response rates in >50% of patients with metastatic BRAF*^V600E^* mutated melanoma (8, 9), and only 5% of metastatic CRC patients with the same BRAF*^V600E^* mutation responded to monotherapy (10, 11). In contrast to melanoma, it is hypothesized that a major factor underlying the lack of clinical response with single-agent BRAF or MEK inhibitor in CRC is a robust adaptive feedback signaling that leads to reactivation of MAPK signaling, often mediated by epidermal growth factor receptor (EGFR) following BRAF-inhibitor treatment (12, 13).

In this case report, we report a patient who had progressive disease after failure of standard chemotherapies in 2017. At this timepoint, the currently approved doublet targeted therapy with encorafenib plus cetuximab, which was approved by the European Medicines Agency (EMA) in 2020, was still under investigation in the BEACON trial, and an off-label use was not possible (14). Due to a lack of therapy alternatives, the patient was offered an off-label use of cetuximab plus dabrafenib plus trametinib based on a few clinical trial reports, which are summarized in the following.

Combined inhibition of BRAF and MEK with dabrafenib and trametinib showed improved response and survival rates compared with dabrafenib alone in metastatic BRAF*^V600E^* mutated melanoma, which resulted in its approval in 2014 (15). However, this combination was only evaluated in a small sample size in metastatic BRAF^V600E^ mutated CRC. In a pharmacodynamic cohort study, a total of 43 patients with BRAF^V600E^ mutated CRC were treated with dabrafenib plus trametinib and showed an overall response rate (ORR) of 12% including a complete response (CR) in one patient and stable disease in further 56% of patients (16). The median progression-free survival (PFS) was 3.5 months. One patient had a CR by week 32 of the study treatment with a duration of response >36 months. Mutational analysis revealed that the patient achieving a CR and two of three evaluable patients achieving a partial response had PIK3CA mutations. Further, the tumor of the patient with CR was microsatellite instable (MSI). To achieve greater MAPK suppression and improved efficacy in patients with metastatic BRAF*^V600E^* mutated CRC, a clinical phase I study with three arms evaluated dabrafenib plus trametinib plus panitumumab versus dabrafenib plus panitumumab versus trametinib plus panitumumab in 142 patients and demonstrated ORR in 21%, 10%, and 0% (17). Median PFS was 4.2, 3.5, and 2.6 months, and median overall survival (OS) was 9.1, 13.2, and 8.2 months. One patient in the triplet and doublet treatment groups (dabrafenib plus panitumumab) had a CR. Analysis of the microsatellite status showed a trend toward a statistically significant increase in PFS in MSI versus microsatellite stable (MSS) tumors. None of the MSS patients remained in the study longer than 1 year with this combination therapy. In the MSI cohort, one patient achieved a partial response lasting >24 months, and another patient had a CR over 26 months. Nevertheless, one patient treated with dabrafenib plus panitumumab was MSS and achieved a CR. Due to the small sample size and a limited number of studies, this targeted combination is not approved in BRAF^V600E^ mutated CRC. Currently, doublet therapy with encorafenib plus cetuximab is the only approved targeted therapy in this patient population from second-line therapy based on the results from the phase III BEACON trial (14).

We want to highlight in this case the potential of targeted therapies in some patients with pretreated, advanced colon cancer and that treatment can be discontinued as an ongoing response. Furthermore, in the Discussion section, EMA-approved standard treatments for metastatic BRAF^V600E^ mutated CRC are summarized, and current areas of research to enhance efficacy and to individualize therapy in different subgroups of metastatic BRAF^V600E^ mutated CRC will be discussed.

## Case description

**Figure 1:**
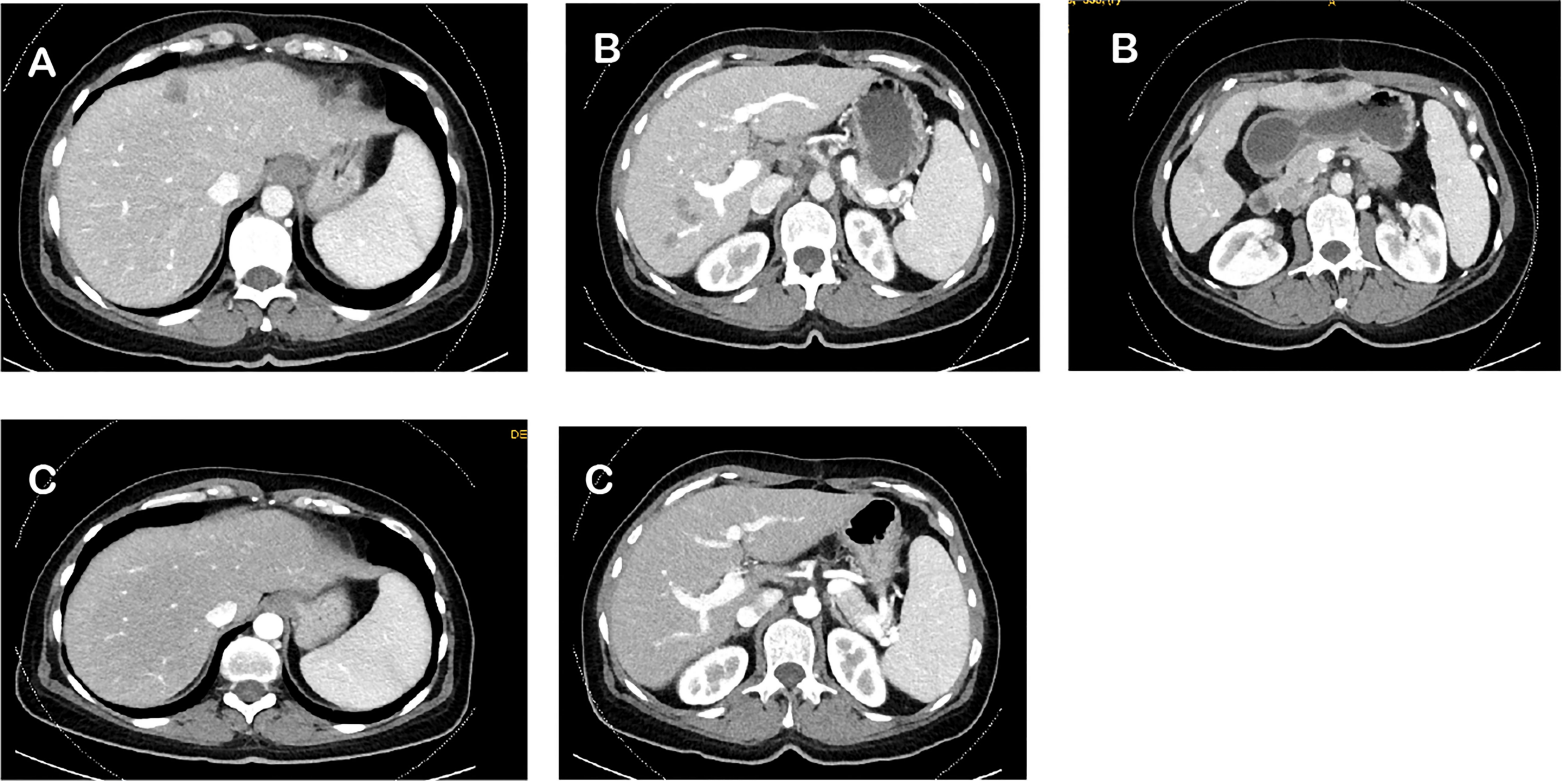
Computed tomography scan (CT scan) regarding the clinical response during whole course of treatment. **(A)** Baseline CT scan in February 2017. **(B)** CT scan after adjuvant CAPOX in June 2017. **(C)** CT scan after 1 year of dabrafenib, trametinib, and cetuximab in October 2018.

A 52-year-old woman without a significant medical history presented to the hospital due to a 3-day history of obstipation, abdominal pain, and nausea in February 2017. On examination, her abdomen was distended and mildly tender on the left side. Blood tests revealed anemia. On the computer tomography scan (CT scan), one suspicious lesion in the liver with a diameter of 3 cm and a suspicious mass in the colon descendens were described (**Figure 1**). In the diagnostic colonoscopy, a 5-cm non-obstructive tumor in the colon descendens was found. Biopsies of the primary tumor confirmed the diagnosis of adenocarcinoma of the colon. In the magnetic resonance imaging of the liver, two suspicious lesions in segments VII and VI were described. The liver metastases were classified by the liver surgeon as primary resectable. An initial hemicolectomy with simultaneous atypical liver resection was performed in February 2017. The histology of the primary tumor revealed a poorly differentiated, MSS, Her2-negative, and BRAF*^V600E^* mutated adenocarcinoma of the colon with a lymphatic vessel and perineural involvement as well as lymph node involvement in eight of 14 removed lymph nodes. The liver metastases were completely resected, and the liver lesions were confirmed histologically to be metastatic lesions. FoundationONE^®^ analysis of the primary tumor showed BRAF*^V600E^* mutation, PTEN-loss, DDR1 R514C alteration, KDM5A R782Q alteration, TP53 Y234 alteration, and MSS status. The tumor mutational burden was 0 Muts/Mb.

A 6-month course of postoperative, pseudoadjuvant chemotherapy with capecitabine and oxaliplatin (CAPOX) was planned. The rationale for pseudoadjuvant chemotherapy with CAPOX was to reduce the risk of recurrences, which occur in approximately 50% of patients with resectable liver metastases. However, the best postoperative strategy for primary resected colorectal liver metastases is uncertain—both pseudoadjuvant chemotherapy and perioperative chemotherapy tend to show a favorable effect in PFS, but not in OS (18–20). Further, the patient preferred an oral regimen. After 3 months of CAPOX therapy, an interim CT scan was performed in June 2017. The CT scan showed five new liver metastases without further metastases in other organs (**Figure 1**), and the tumor marker carcinoembryonic antigen (CEA) was elevated. A first-line palliative chemotherapy regimen with FOLFIRI and bevacizumab was administered from June until September 2017. After 3 months, the CT scan showed further progress in the liver, and tumor markers were further increasing. Resectability of the liver metastases was excluded. For second-line therapy, the patient was randomized in the control arm of the BEACON study, and FOLFIRI plus cetuximab was administered for 2 months in this trial. The interim CT scan in November 2017 showed progression of the liver metastases and detection of new metastases in the lung, and retroperitoneal lymph nodes metastases and tumor makers further increased. According to the study, the patient went off protocol due to progressive disease. A cross-over to one of the targeted-treatment arms in the BEACON study or off-label use of this targeted therapy was not possible.

**Figure 2:**
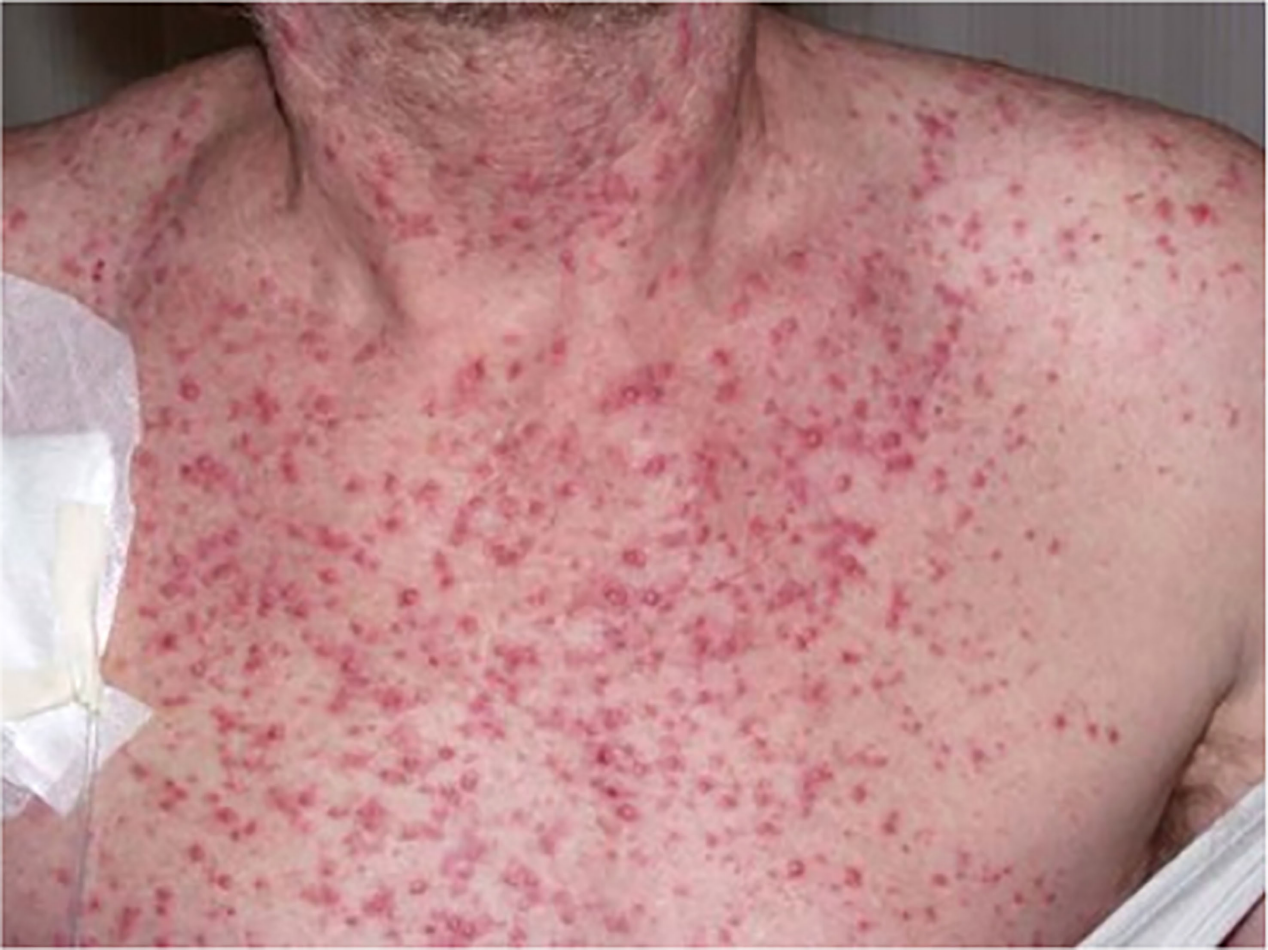
Pronounced skin toxicity due to cetuximab therapy.

**Figure 3:**
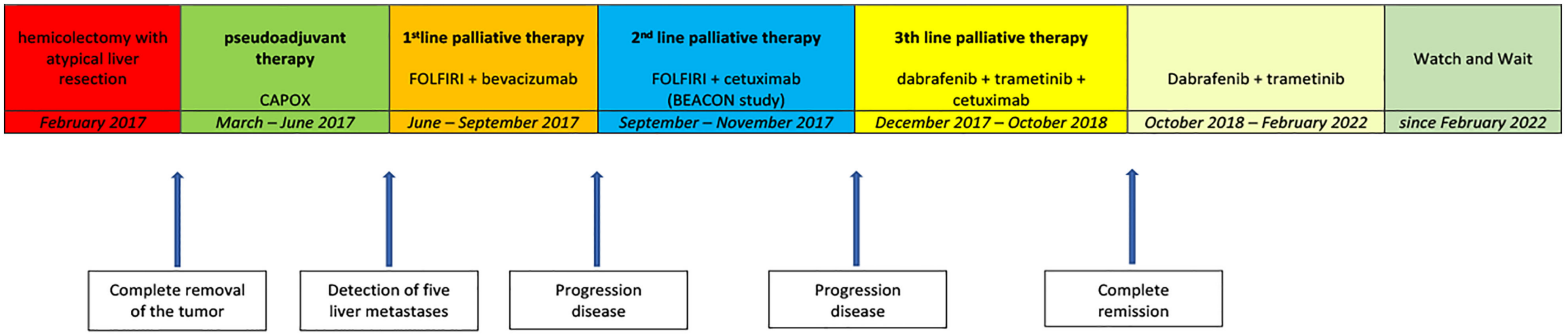
Flowchart of the whole course of treatment.

**Figure 4:**
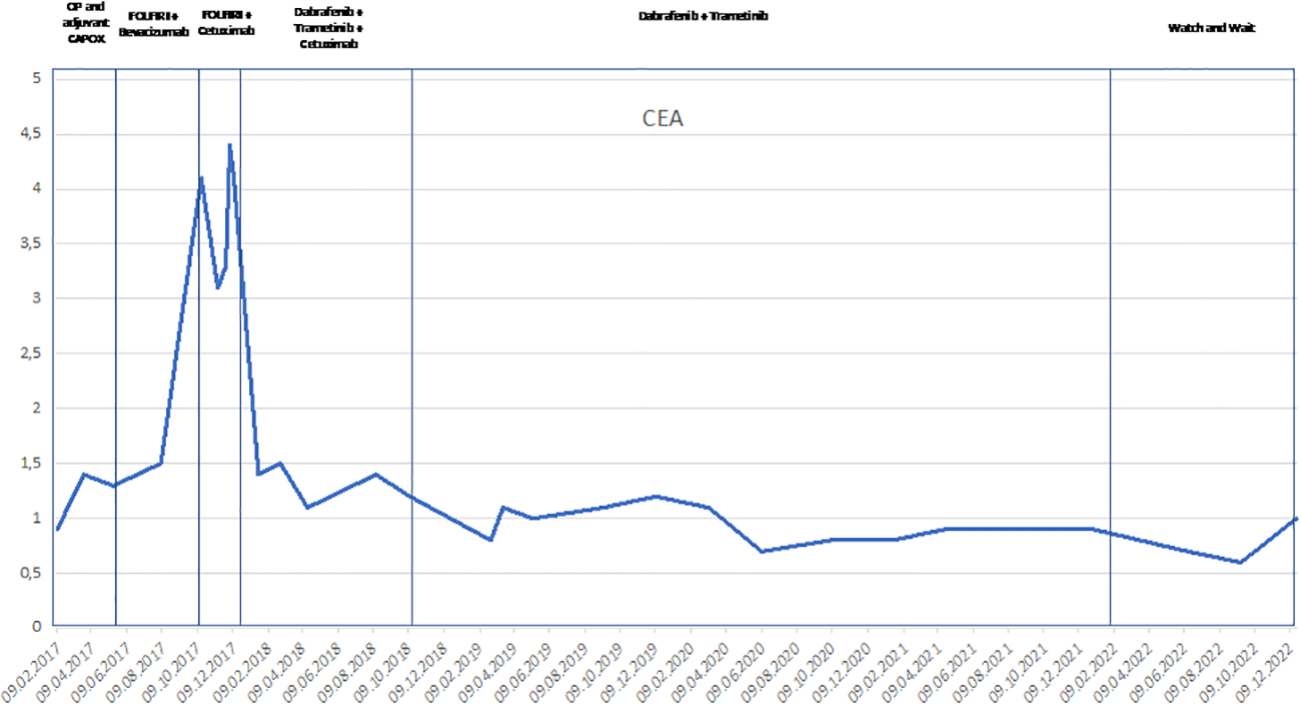
Line chart of the changes of the tumor marker CEA during the process of the treatment since February 2017. CEA, carcinoembryonic antigen.

The performance status was reduced to Eastern Cooperative Oncology Group (ECOG) performance status 2 due to the progressive disease, but the patient was willing to receive further therapy. Because of the lack of promising third-line therapy in BRAF*^V600E^* mutated CRC, the patient received an off-label use of dabrafenib, trametinib, and cetuximab based on reports of a few clinical phase I–II trials, which was mentioned above (16, 17). The therapy was started in December 2017. Two months after the beginning of the third-line palliative therapy, the CT scan showed partial response in the liver, lung, and retroperitoneal lymph nodes. After another 2 months of therapy, the lung metastases and retroperitoneal lymph node metastases could no longer be detected on the CT scan. The liver metastases had almost disappeared. Due to skin toxicity with papulopustular eruption grade 3 (**Figure 2**), steroid-containing cream and 100 mg of minocycline per day were prescribed, and cetuximab therapy was temporarily stopped. Furthermore, the patient suffered from recurrent urinary tract infections due to mucosal toxicity requiring antibiotic therapy in the early stages to prevent urosepsis. In August 2018, no further progression was detected on the CT scan (**Figure 1**), and in October 2018, a PET/CT showed a CR. Since October 2018, cetuximab was terminated due to persistent severe skin toxicity and recurrent urinary tract infections, and double therapy with dabrafenib and trametinib was continued with better tolerance. The urinary tract infections were fewer, and the skin recovered. Therapy with dabrafenib and trametinib was terminated on February 2022 at the request of the patient, and a watch-and-wait strategy with CT scan and blood tests including CEA every 3 months was recommended. Until January 2023, the patient is still in CR and in excellent general condition. **Figure 3** shows an overview of the whole course of treatment in this patient, and **Figure 4** shows the changes in the tumor marker during the therapy. The patient consented to the publication of her medical history.

## Discussion

To the best of our knowledge, achieving an ongoing CR after treatment cessation with dabrafenib, trametinib, and intermittent cetuximab as third-line treatment in a patient with an advanced BRAF*^V600E^* mutated, MSS colon cancer is unique.

### State-of-the-art therapy

The first-line recommendations for patients with metastatic BRAF*^V600E^* mutated CRC are FOLFOXIRI or doublet chemotherapy regimen plus bevacizumab based on the subgroup analysis of the TRIBE study (21) and TRIBE 2 study (22, 23). The decision to use triplet or doublet chemotherapy regimens plus bevacizumab should be based on a risk/benefit discussion with the patient. In 2020, EMA approved doublet therapy with encorafenib + cetuximab for the treatment of patients with BRAF^V600E^ mutated metastatic CRC (mCRC) who have received prior systemic therapy, according to the results of the phase III BEACON trial (14). In this trial, 665 patients were randomized to receive triplet therapy with encorafenib plus binimetinib plus cetuximab or doublet therapy with encorafenib plus cetuximab or standard therapy with FOLFIRI/irinotecan plus cetuximab. The median PFS and the median OS for triplet and doublet therapies were superior compared to those of the standard group (median PFS 4.3 *vs.* 4.2 *vs.* 1.5 months; median OS 9.0 *vs.* 8.4 *vs.* 5.3 months). The ORR was 26% *vs.* 20% *vs.* 2%. However, the study was not powered to compare the two experimental groups directly. However, descriptive analyses comparing triplet and doublet arms showed similar efficacy in the overall population across endpoints including PFS and OS, and adverse events were higher with triplet compared to doublet therapies. The results suggested that the doublet regimen is sufficient to maximize the OS benefit with better tolerability, and doublet therapy was approved by EMA. Later-line therapies include other chemotherapy combinations, TAS-102, and/or regorafenib with modest effectiveness (24).

For metastatic BRAF*^V600E^* mutated CRC with MSI-h, the therapeutic approach is different, and microsatellite status should be tested up-front. In a pooled analysis of four studies, the incidence of BRAF*^V600E^* mutated CRC was 34.6% in patients with mismatch repair deficiency and 6.8% in patients with microsatellite-stable CRC (25). The molecular relationship between BRAF mutation and MSI is through high-level CpG island methylator phenotype and MLH1 promotor methylation. Pembrolizumab is approved by the EMA for patients with metastatic MSI-h CRC in the first-line setting and after fluoropyrimidine-based combination therapy based on the results from the Keynote-177 study (26) and Keynote-164 study (27). The Keynote-177 study showed that pembrolizumab was superior in terms of PFS and OS compared with chemotherapy in the overall MSI-h population as well as in patients with BRAF*^V600E^* mutated CRC and MSI-h (26).

In second-line and third-line settings, pembrolizumab showed highly promising outcomes with ORR of 20% and 55% in patients with BRAF*^V600E^* mutated CRC and MSI-h (27). Furthermore, in the CheckMate-142 trial, the combination of nivolumab plus ipilimumab in MSI-h-patients who received prior chemotherapy showed an ORR of 55% and a 12-month OS rate of 85%, irrespective of BRAF status (28).

BRAF*^V600E^* mutated CRC is not a homogenous disease, and up-front treatment decision is currently made by microsatellite status. From the second line of therapy, targeted therapy represents the standard of care and significantly improved outcomes. Nevertheless, the prognosis of metastatic BRAF*^V600E^* mutated CRC remains poor, and further investigations are needed to improve survival.

### Areas of research

The current objectives of the research are a) the implementation of targeted therapies in the first-line setting and b) combining targeted therapies with chemotherapy or c) immunotherapy or d) other targeted therapies based on molecular analyses. Further, there is a great need to predict the outcomes by identification of e) different molecular subgroups.

a. The ANCHOR study evaluated in a single-arm phase II study encorafenib plus binimetinib plus cetuximab in previously untreated metastatic BRAF^V600E^ mutated CRC, and the results were recently published (29). Among 95 patients, the ORR was 47.4% with all partial responses. The median PFS was 5.8 months, and the median OS was 18.3 months. The primary endpoint was met. However, these results showed that the combination therapy in the first-line setting is quite similar to the recommended chemotherapy-based regimens in the first-line setting of metastatic BRAF^V600E^ mutated CRC. The results signal that there is a need to evaluate mechanisms of acquired resistance, as the short PFS interval is likely due to resistance that arises despite inhibiting BRAF, MEK, and EGFR.
b. To improve the outcome, the phase III BREAKWATER study explores in three arms the combination of encorafenib plus cetuximab with or without chemotherapy (mFOLFOX or FOLFIRI) versus control (mFOLFOX, FOLFIRI, and FOLFIRINOX ± bevacizumab) in the first-line setting in 765 patients (ClinicalTrials.gov Identifier:NCT04607421). Updated safety and anti-tumor activity data from the BREAKWATER safety lead-in demonstrated that the addition of chemotherapy to encorafenib plus cetuximab was generally tolerable with preliminary promising antitumor activity (30). The final results are eagerly awaited.

c. A further interesting approach is the combination of immunotherapy and targeted therapy in metastatic BRAF^V600E^ mutated CRC. Currently, immunotherapy is only approved in patients with MSI-h. However, in metastatic BRAF^V600E^ mutated CRC, the addition of immunotherapy is evaluated in not only MSI-h patients but also MSS patients based on data from preclinical studies that suggest that combining MAPK inhibition and immunotherapy could enhance antitumor efficacy in BRAF and KRAS mutant cancers (31–33). A recent proof-of-concept single-arm phase II study evaluated the addition of a PDL-1 inhibitor spartalizumab to dabrafenib and trametinib in patients with BRAF^V600E^ mutated CRC (34). Of the 37 included patients, most of them were MSS (n = 32). In these patients with MSS BRAF^V600E^ mutated CRC, the ORR was 25%, and the disease control rate was 75%. Median PFS was 5 months with 18% of patients remaining on therapy for over 1 year. The authors of the study suggest a potential tumor cell-intrinsic mechanism of synergy between MAPK inhibition and immunotherapy, and additional studies are needed to more fully understand the benefits of MAPK inhibition combined with immunotherapy in MSS BRAF^V600E^ mutated CRC. A phase II study evaluates the addition of nivolumab to encorafenib plus cetuximab versus doublet therapy with encorafenib plus cetuximab in BRAF^V600E^ mutated, MSS CRC after the failure of at least one prior treatment. The primary endpoint is PFS (ClinicalTrials.gov Identifier: NCT05308446). The SEAMARK trial investigates in a phase II clinical trial the efficacy of encorafenib plus cetuximab plus pembrolizumab versus pembrolizumab alone in patients with untreated metastatic BRAF^V600E^ mutated CRC and MSI-h (ClinicalTrials.gov Identifier: NCT05217446).
d. The mechanism of resistance to targeted therapies is not completely understood. Unlike other tumors with BRAF^V600E^ mutations, like melanoma, non-small cell lung cancer, and papillary thyroid cancer, BRAF mono-inhibition in CRC resulted only in marginal clinical activity. BRAF inhibition causes a rapid feedback activation of EGFR because of the missing negative feedback mechanism driven by ERK1/2 activation and leads to MEK1/2 activation through several escape mechanisms. Various mechanisms of resistance have been discovered, from activation of various receptor tyrosine kinases to activation of other cell signaling pathways such as the PI3K/AKT pathway (35, 36). Receptor tyrosine kinases have multiple pathways by which they can promote cell signaling, and reactivation of receptor tyrosine kinases following inhibition of the MAPK pathway stimulates cellular growth through various pathways. The majority of resistances are centered around the reactivation of the MAPK pathway. Several analyses of mutational profiles and preclinical studies suggested activations of the phosphoinositide 3-kinase (PI3K) pathway as a potential mechanism of resistance to BRAF inhibitors (37). To overcome the potential mechanism of resistance, the combination PI3K inhibitor alpelisib was investigated (38) in 28 refractory BRAF^V600E^ mutated CRC in a phase Ib study and showed good tolerability of the triplet therapy but with quite similar efficacy compared with dual therapy. The ORR was 18%, and the disease control rate was 93% in the triplet arm. However, this was a small study. In a subsequent phase II study, 52 patients received the same regimens and demonstrated a PFS of 5.4 versus 4.2 months in the triplet versus doublet therapy (39). PTEN loss or the signaling pathway STAT has also been associated with intrinsic resistance to BRAF/MEK targeted therapies. Targeting the Wnt/β-catenin signaling pathway represents another potential future treatment option, as Wnt was shown to activate signaling through RAF-MEK-ERK targeting (40). With further understanding of the complex mechanism of resistance, the therapeutic landscape will be changing to individualize therapy strategies based on molecular subtypes, and studies are needed to investigate multi-targeted combination treatments to overcome resistance.
e. A recently published study evaluating whole-exome sequencing identified inactivating mutations in RNF43, a negative regulator of WNT, to predict improved response rates and survival in patients with BRAF^V600E^ mutated CRC and MSS tumors treated with anti-BRAF/EGFR combination therapies (41). The RNF43 mutation frequency was approximately 43%–44% (92%–100% in the MSI cohort and 28%–30% in the MSS cohort) in the discovery and validation cohort. The ORR in the RNF43^mutated^ subgroup was 63% compared with 31% in the RNF43^wild-type^ subgroup. Patients with the MSS-RNF43^mutated^ subtype achieved the highest ORR with 54% compared to the MSS-RNF43^wild-type^ subtype (21%) and MSI- RNF43^mutated^ subtypes (18%). Evaluation of circulating tumor DNA (ctDNA) is a further area of research. In an exploratory analysis of the BEACON trial, ctDNA was measured at baseline and the end of treatment. Variant allele frequency (VAF) of BRAF*^V600E^* was measured, and patients were grouped in high and low categories (BRAF*^V600E^* or ctDNA was not detected). Over 90% of patients had detectable BRAF*^V600E^* mutations in the ctDNA. Patients with a higher VAF for BRAF*^V600E^* had a worse prognosis. Compared with the control group of the BEACON trial, patients with triplet or doublet therapy had increased response rates, independent of VAF. CtDNA VAF was found to be prognostic but not predictive of drug response (42). Biomarker analysis of the VELOUR (43) and RAISE studies (44) indicated a non-significant benefit of the addition of aflibercept in the VELOUR study and ramucirumab in the RAISE study to chemotherapy in BRAF*^V600E^* mutated mCRC compared with wild-type BRAF mCRC. Prognostic and predictive biomarkers are of great interest to further individualize therapy in this rare subgroup of metastatic CRC.

## Conclusion

Patients with BRAF^V600E^ mutated, MSS tumors have the worst prognosis among the variety of subgroups of CRC patients. The treatment options for patients with BRAF^V600E^ mutated CRC are limited. Our patient case showed that even in later lines, a targeted therapy combination could achieve an ongoing complete remission. Even a de-escalation from triplet to doublet therapy and subsequent discontinuation of therapy showed ongoing CR in this impressive patient case.

## Data availability statement

The original contributions presented in the study are included in the article/supplementary material. Further inquiries can be directed to the corresponding author.

## Ethics statement

Written informed consent was obtained from the individual(s) for the publication of any potentially identifiable images or data included in this article.

## Author contributions

All authors listed have made a substantial, direct, and intellectual contribution to the work and approved it for publication.
